# Deacetylation Ensures Timely Regulation of Flowering

**DOI:** 10.1371/journal.pbio.1001646

**Published:** 2013-09-03

**Authors:** Amy Coombs

**Affiliations:** Freelance Science Writer, Chicago, Illinois, United States of America

[Fig pbio-1001646-g001]If a plant flowers too late in the season, the cold temperatures will prevent fruit from ripening. Similarly, if trees bloom at the first sign of spring, pollinating insects might not be around to fertilize their flowers. This is in part why plants use complex regulatory pathways to interpret environmental signals that determine when they flower. For example, some plants bloom after exposure to longer periods of daylight, but this can also trigger the regulatory pathways thought to repress precocious flowering.

**Figure pbio-1001646-g001:**
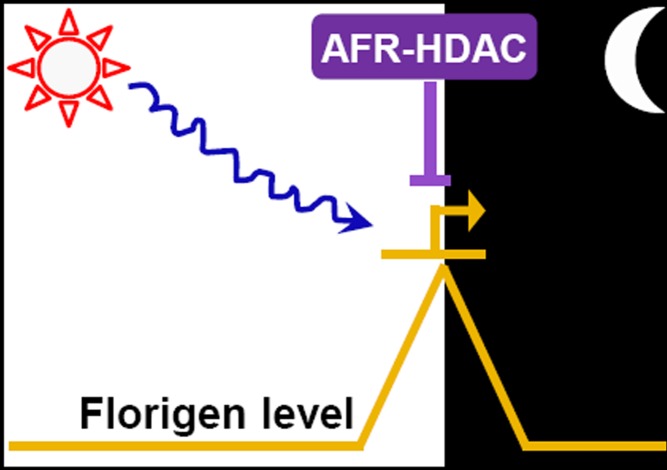
The exposure of *Arabidopsis* plants to longer periods of daylight results in the transcriptional activation of a florigen gene specifically at the end of a day. A histone deacetylase complex (AFR-HDAC) “checks and balances” this activation to prevent overproduction of this flowering signal and thus precocious blossoming. Image credit: Yuehui He.

As to how plants regulate such competing signals, research now points to a histone deacetylation pathway that dampens the expression of a molecule thought to control flowering. These findings, published in the August issue of *PLOS Biology*, identify a new protein that is integral to the plant histone deacetylase complex.

Histone deacetylation represses gene transcription by removing acetyl groups from the tails of core histones; this deacetylation process requires a complex of proteins to house the deacetylase enzyme and to ensure it acts on the correct targets. Sin3-HADC is one of the best-characterized deacetylase complexes—it includes the RPD3 deacetylase enzyme, the Sin3-HDAC scaffold, and structural proteins SAP18 and SAP30. The complex is found in yeast, although mammalian homologs also exist. In plants, a SAP18 homolog is known to impact the formation of flower organs, and numerous RPD3 and scaffold homologs have been identified. However, the absence of a SAP30-like protein in Arabidopsis and in other plant models has raised questions about the role of a Sin3-like complex in plant gene regulation.

To investigate, Yuehui He from the National University of Singapore and his colleagues searched the Arabidopsis protein database for a functional relative of SAP30. As prior research found no plant homolog to the full yeast protein sequence, the team searched for a 30-amino acid binding region that links SAP30 to the Sin3-HADC complex. They identified two proteins, AFR1 and AFR2, both of which contain a binding region similar to that of SAP30.

To see whether AFR1 and AFR2 interact with deacetylase complexes, the scientists engineered proteins that fluoresce when bound. Plant homologs of SAP18 and the RPD3 deacetylase enzyme were attached to fragments of enhanced yellow fluorescent protein (EYFP). Complimentary EYFP fragments were also attached to AFR1 and AFR2. After expressing combinations of the proteins in onion cells, the researchers observed fluorescence in the nucleus, confirming that AFR1 and AFR2 can be part of a histone deacetylase complex.

Testing the biological function of the newly discovered proteins led to an even more surprising result—AFR1 and AFR2 appear to mediate histone deacetylation at a key locus for flowering control. The team of investigators disrupted the transcription of both AFR1 and AFR2 in Arabidopsis by using insertional transfer-DNA. The double loss of function mutants had greater amounts of the acetylated histone H3 than plants capable of expressing AFR1 and AFR2, suggesting both proteins are involved in deacetylation and subsequent gene repression.

Chromatin immunoprecipitation assays located AFR1 and AFR2 proteins to the *FT* locus, which encodes a major component of the mobile flowering signal called florigen. AFR1 and AFR2 bound directly to the *FT* promoter, but only at the end of long days of sunshine. But in the middle of the day, or after a short day of light, neither protein localized to the *FT* site.

In Arabidopsis, florigen is produced by the leaf after a plant has been exposed to a long day of sunlight. It then travels from the leaf to the shoot apical meristem—a population of rapidly dividing cells located at the tip of plant shoots that produce leaves and flowers—where it induces flowering. As early flowering is sometimes disadvantageous, plants need a mechanism to curb and control florigen production. He and his colleagues predicted that AFR1 and AFR2 might offer one such mechanism, and they designed a series of experiments to compare *FT* levels between AFR protein mutant and normal plants.

These experiments revealed that *FT* transcript levels increased at dusk in plants that lack AFR1 and AFR2, while normal plants had lower levels of FT. The researchers also observed that the expression of AFR1 and AFR2 peaks at the end of a long day of sunlight—the time when florigen levels are high and the flowering signal is strong. The researchers' findings also suggest that AFR1 and AFR2 are recruited to the FT locus by two transcription factors, AGL18 and AGL15, which are thought to be involved in FT repression and flowering inhibition. This suggests the newly discovered AFR proteins delay flowering by dampening the level of FT signal at the end of long days. Indeed, after being exposed to an extended period of light, normal plants flowered later than those that lacked AFR1 and AFR2.

These findings are among the first to outline a detailed mechanism by which histone deacetylation regulates when plants flower in response to light exposure. As AFR1 and AFR2 were unknown prior to this study, earlier investigations could not outline the full signaling pathway involved in the regulation of this important light response. This work now provides a missing piece of the puzzle, and demonstrates how transcriptional repression by histone deacetylation helps plants to flower at just the right time.

**Xiaofeng G, Wang Y, He Y (2013) Photoperiodic Regulation of Flowering Time through Periodic Histone Deacetylation of the Florigen Gene *FT*** **. doi:10.1371/journal.pbio.1001649**

